# Optical characteristics of refractive-index-matching diffusion layer in organic light-emitting diodes

**DOI:** 10.1038/s41598-019-45000-w

**Published:** 2019-06-18

**Authors:** Cheol Hwee Park, Jae Geun Kim, Sun-Gyu Jung, Dong Jun Lee, Young Wook Park, Byeong-Kwon Ju

**Affiliations:** 1 Display and Nanosystem Laboratory, School of Electrical Engineering, Korea University Seoul, Seoul, 136-713 Republic of Korea; 2 School of Mechanical and ICT Convergence Engineering, SUN MOON University, Chungcheongnam-do, 31460 Republic of Korea

**Keywords:** Electrical and electronic engineering, Organic LEDs, Electrical and electronic engineering, Organic LEDs

## Abstract

We developed a diffusion layer with a refractive index-matching layer added to a transparent nanoscale polymer-based structure to obtain its effective scattering effects. The diffusion layer had higher haze when the refractive index-matching layer to a higher refractive index was used. This diffusion layer was applied to conventional organic light-emitting diodes (OLEDs) and micro-cavity OLEDs (MC-OLEDs) to evaluate the characteristics. When a diffusion layer was applied to conventional OLEDs, the external quantum efficiency (EQE) was 31.1% higher than that of the device without a diffusion layer due to the reduction of the substrate mode, and the viewing angle characteristic was also improved. Then, when the diffusion layer was applied to the MC-OLEDs, all devices showed similar EQE values regardless of the presence or absence of the diffusion layer, and the viewing-angle-dependent characteristics were greatly improved by the diffusion layer. Furthermore, when the diffusion layer was used with polarizer film, the black color implementation was not affected by the polarizer film, proving that it is applicable to actual OLED display products.

## Introduction

Organic light-emitting diodes (OLEDs) are gaining popularity as next-generation displays and lighting taking advantage of their low thickness, self-luminous properties, high color purity and gamut, fast response speed, and various applicable characteristics like flexibility and foldability^1–4^. The light-extraction technology among various research issues of OLEDs is an essential element to obtain high-performance display and lighting. However, there is a problem in that the conventional flat OLEDs can emit only 20% of the light generated in the emitting layer. There are three major reasons for this loss. The first is the total internal reflection (TIR) due to the refractive index difference between each layer. The TIR due to refractive index mismatch is classified into a substrate mode occurring at the air/glass substrate interface and a waveguide mode occurring between the transparent electrode, such as indium tin oxide (ITO), and the glass substrate. Second, there is absorption of light from the material in each layer. Finally, there is a surface plasmon polariton (SPP) mode that occurs at the interface between the reflective electrode and the organic layer^5–7^.

To overcome these causes of light loss, many research groups have studied light-extraction technology. Representative methods include randomly distributed structures^8,9^, photonic crystals^10,11^, scattering layers^12,13^, low-refractive-index grids^14^, micro lens array (MLA)^15–17^, patterned substrate surfaces^18^, corrugated structures^19,20^, high-refractive-index substrates^21^, and micro-cavity structures^22–24^. Although these light-extraction techniques have been reported to improve the efficiency of OLEDs, they have the disadvantages that the fabrication steps are complex and difficult and that they are difficult to apply to actual displays and lighting devices. Particularly, there are fatal drawbacks in that the light-extraction techniques applied inside devices can affect the electrical characteristics of the devices. Of these technologies, MLA and micro-cavity structure are used as relatively simple and effective light-extraction techniques. The MLA can improve the efficiency of the device effectively and stably, but because the size of the lenses for MLA is several tens of micrometers, MLA is difficult to apply to high-resolution displays with small pixels. Koh *et al*. reported that they fabricated green OLED device which show 65% and 77% enhancement of external quantum efficiency and power efficiency respectively by attaching nanoporous polyimide film^25^. However, nanoporous polyimide film has low transmittance of less than 60% in the visible light wavelength range. Therefore, it is necessary to develop a sufficiently transparent external light-extraction structure capable of effectively improving the external light efficiency while remaining nanoscale and analyze the characteristics of OLED such as viewing angle and contrast-ratio loss for commercialization. It is possible to manufacture the micro-cavity structure through a relatively simple process and improve the efficiency of the device. In addition, the micro-cavity structure has features that can enhance the color purity by narrowing the electroluminescence (EL) spectrum. However, these micro-cavity structure has the drawback of dependence of the color and luminance on the viewing angle.

In this paper, we fabricated a refractive-index-matching diffusion layer through a simple process. The diffusing layer consists of a nanoscale structure and a refractive-index-matching layer deposited on the structure for higher scattering effects. The fabricated diffusion layer has a high transmittance of 85% or more and a maximum haze of 30% at a wavelength of 540 nm according to the refractive index of the refractive-index-matching layer. We analyzed the efficiency and viewing-angle characteristics of the device by attaching these diffusion layers to conventional OLEDs with a transparent electrode and a micro-cavity OLEDs (MC-OLEDs) with a micro-cavity structure.

## Results and Discussion

### Fabrication and optical properties of diffusion layer

**Figure 1:**
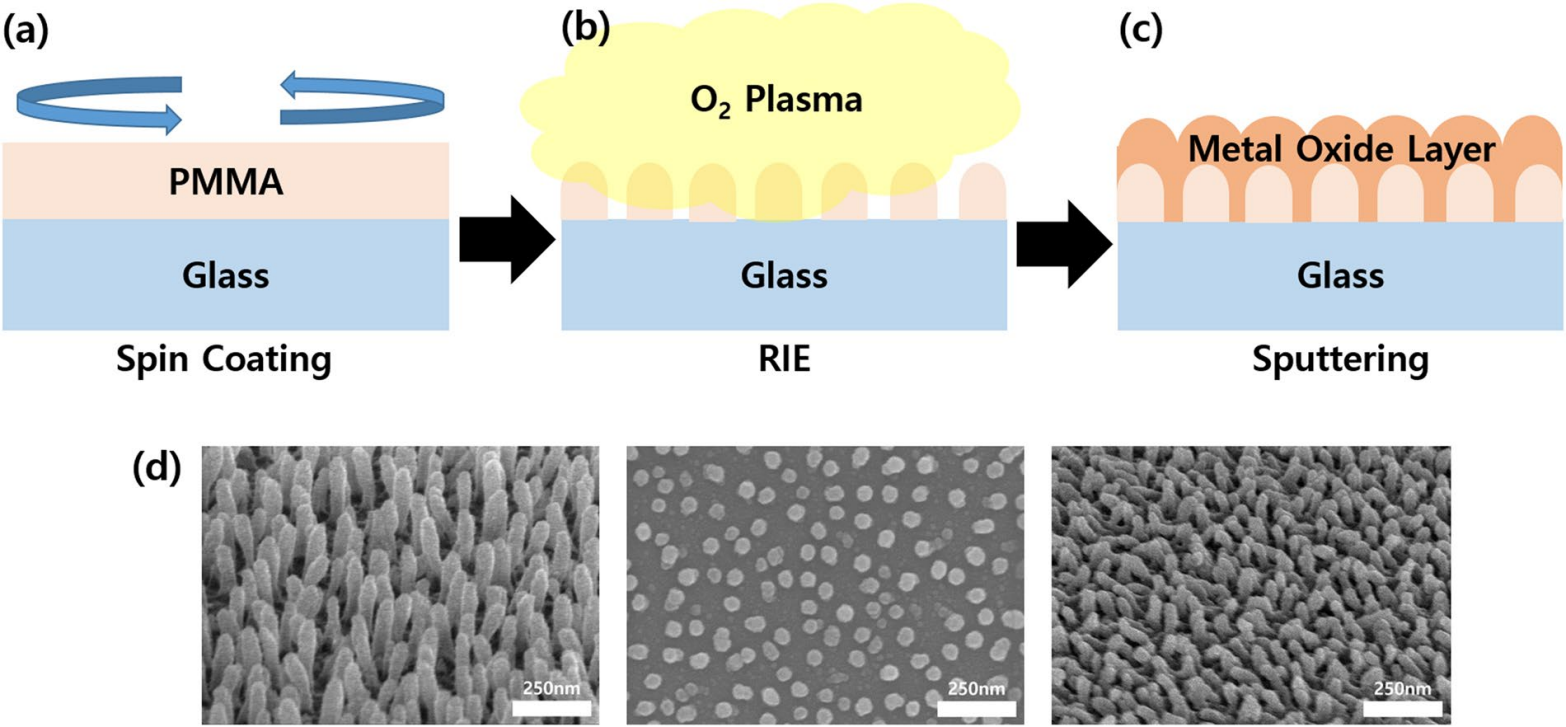
Fabrication process of diffusion layer: (**a**) PMMA spin coated on the substrate, (**b**) etching the PMMA by reactive ion etching (RIE) O_2_ plasma, and (**c**) deposition of the refractive matching layer. (**d**) FE-SEM images of the diffusion layer (left: tilted PMMA nanostructure, middle: plane PMMA nanostructure, right: diffusion layer).

Figure 1 shows the fabrication process of the diffusion layer and the field-emission scanning electron microscopy (FE-SEM) images of the fabricated diffusion layer. First, poly(methyl methacrylate) (PMMA) was spin coated on the cleaned glass substrate at a rotational speed of 1000 rpm. It was then heated on a 170-°C hot plate for 40 min to cure the PMMA. In the next step, O_2_-gas plasma etching was performed through reactive ion etching (RIE) to form PMMA nanoscale pillar structures^26,27^. The radio-frequency (RF) power used for the plasma etching was 200 W, and the plasma etching proceeded for 2 min. Finally, a metal-oxide material with a different refractive index from PMMA (n = 1.5) was deposited 70 nm as a refractive-index-matching layer by RF sputtering on the PMMA structures, as shown in the left image of Fig. 1(d). Aluminum oxide (Al_2_O_3_) and zinc oxide (ZnO), which have higher refractive indices than PMMA, were used as the refractive-index-matching layer. The surface of the refractive-index-matching layer on the PMMA structure has a corrugated structure, as shown in the right image of Fig. 1(d), due to the shape of the PMMA structures. The type of diffusion layers thus fabricated is classified according to the materials of the refractive-index-matching layer in the upper layer.

**Diffusion layer 1**: Only PMMA structure (without refractive-index-matching layer)

**Diffusion layer 2**: PMMA structure + Al_2_O_3_ refractive-index-matching layer

**Diffusion layer 3**: PMMA structure + ZnO refractive-index-matching layer

**Figure 2:**
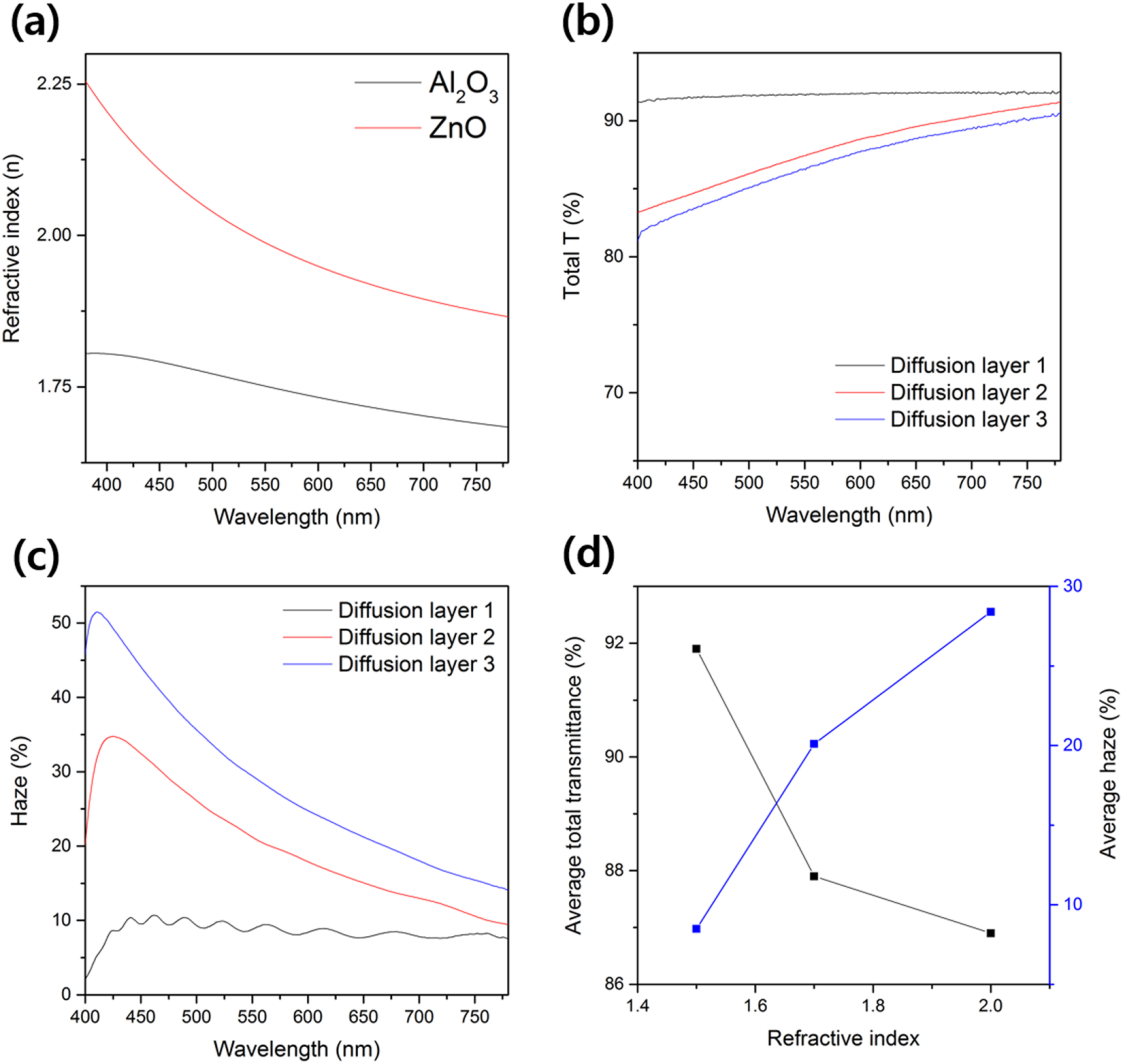
(**a**) Refractive indices of Al_2_O_3_ and ZnO, (**b**) total transmittance of diffusion layers, (**c)** haze of diffusion layers, and (**d**) average total transmittance and haze according to refractive index of diffusion layers.

The refractive indices of Al_2_O_3_ and ZnO, which are metal-oxide materials used as refractive-index-matching layers, were measured using a thin-film measurement system. As can be seen in Fig. 2(a), Al_2_O_3_ has a refractive index of about 1.75 and ZnO has a refractive index of 2.0 at 540 nm, which is a green emission wavelength region. Because the refractive index is 1.5 for the PMMA structure existing under the refractive-index-matching layer with refractive indices of 1.75 or 2.0, the light path is changed due to the refractive index difference between the two layers, and the scattering effect is caused. To determine the scattering effect due to the refractive index difference between the two layers, we measured the total and diffuse transmittance. As shown in Fig. 2(b), the total transmittance of Diffusion layer 1, comprising only the PMMA structure, is higher than 90% in the visible region because PMMA is a transparent polymer material with a transmittance of 90% or more in the visible region. On the other hand, Diffusion layers 2 and 3, in which Al_2_O_3_ and ZnO are respectively deposited, show lower total transmittance values than Diffusion layer 1. This decrease in transmittance is presumably due to the transmittance of Al_2_O_3_ and ZnO on the top. Nevertheless, it can be seen that all the diffusion layers have a high transmittance of 80% or more in the visible-light region. The total transmittance in the green wavelength region (at 540 nm) used in this paper were 91.9%, 87.2%, and 86.1% in Diffusion layers 1, 2, and 3, respectively. Next, we have calculated the haze value, which indicates the degree of scattering, from the total and diffuse transmittance. The method of calculating the haze is shown in the following equation^28^.

1 $${\rm{Haze}}=\,\frac{Total\,trasmittance-specular\,transmittanc=(diffuse\,transmittance)}{Total\,\,transmittance}$$

As a result of the haze calculation, Diffusion layers 1, 2, and 3 showed a haze of 8.6%, 22.2%, and 30.5% at a wavelength of 540 nm, respectively. It can be seen that Diffusion layer 1, composed only of the PMMA structure, does not cause sufficient light scattering, whereas light scattering occurs effectively in Diffusion layers 2 and 3, to which the refractive-index-matching layer is applied. Furthermore, as the refractive index of the refractive-index-matching layer on the PMMA structure increases (as the refractive index difference increases), the haze tends to increase. The haze along the wavelength tends to increase as the wavelength becomes shorter in the visible light region (toward the blue region). However, in the case of Diffusion layer 1, a uniform haze value appears in the entire visible wavelength region. The average total transmittance and haze of the diffusion layers shown in Fig. 2(d) are summarized as follows, and the total transmittance and haze at 540 nm of the diffusion layers are summarized in Table S1 (Supporting Information, Table S1). As the refractive index of the diffusion layer increases, the average total transmittance decreases but the haze increases.

### Optical properties of diffusion layer in OLEDs

We confirmed the effect of applying the diffusion layers with various refractive indices fabricated in this way to actual OLED devices. First, the diffusion layers were applied to the outside of the OLEDs of the conventional structure using the transparent ITO electrode as the anode, and the characteristics were evaluated. The OLED devices were fabricated sequentially on the opposite side of the glass substrate where the diffusion layer is present. The OLED comprises ITO as the anode, 1,4,5,8,9,11-hexaazatriphenylene-hexacarbonitrile (HATCN) as the hole injection layer, N,N′-Bis(naphthalen-1-yl)-N,N′-bis(phenyl) benzidine (NPB) as the hole-transport layer, Tris-(8-hydroxyquinoline)aluminum (Alq_3_) as the emission and electron-transport layers, lithium fluoride (LiF) as the electron-injection layer, and Al as the cathode. The structure of the OLED devices is shown in Fig. S1 (Supporting Information, Fig. S1). The sampling of the devices according to the type of diffusion layer applied is as follows: **Non-Cavity** (only OLED), **Device A** (OLED with Diffusion layer 1), **Device B** (OLED with Diffusion layer 2), and **Device C** (OLED with Diffusion layer 3).

**Figure 3:**
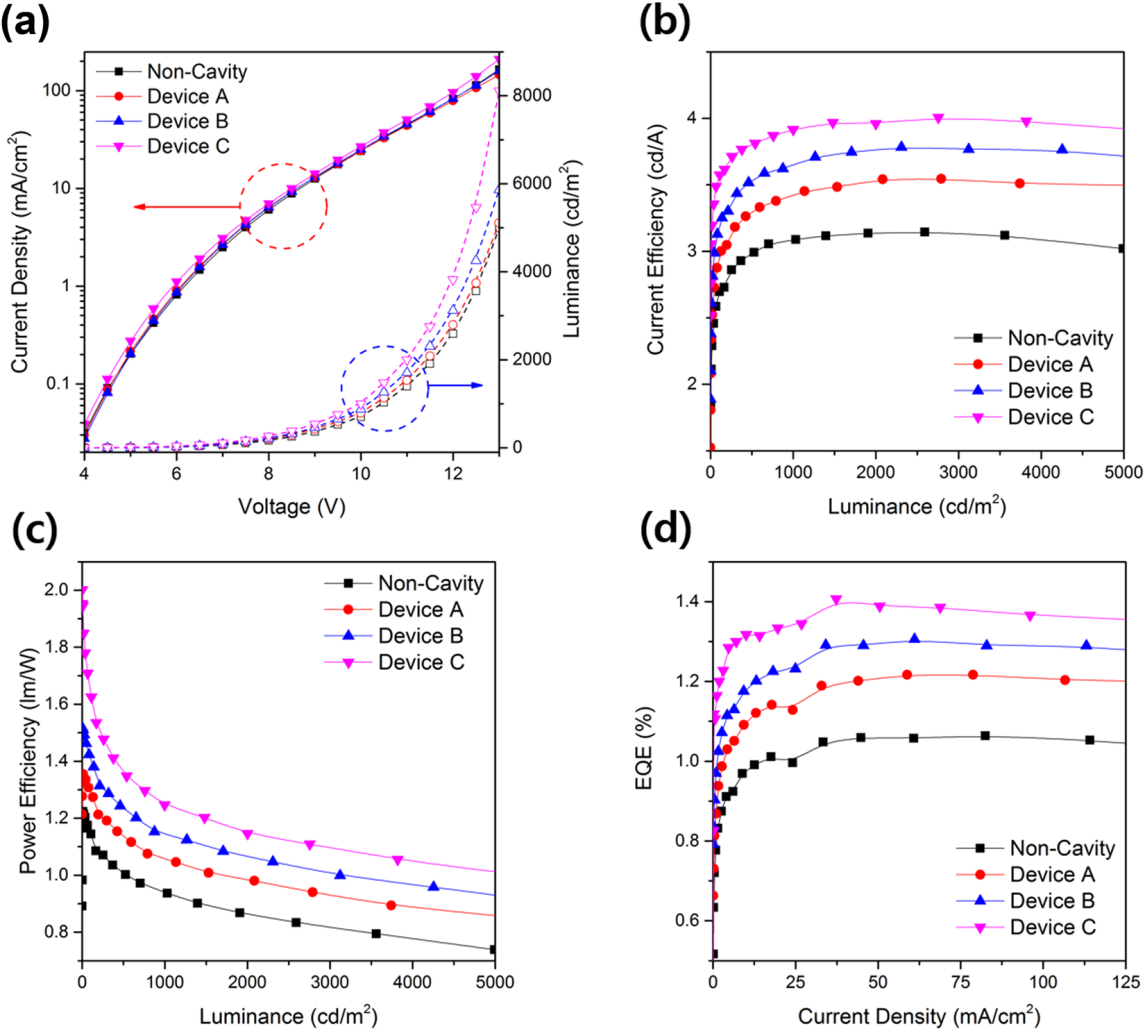
EL characteristics of OLEDs with diffusion layers: (**a**) current-density–voltage–luminance, (**b**) current-efficiency–luminance, (**c**) power-efficiency–luminance, and (**d**) EQE–current-density relations.

Figure 3 shows the EL characteristics of OLEDs with diffusion layers. As for the current density and luminance characteristic according to the voltage, it can be seen that, in the case of the current density, all the devices have nearly the same current-density characteristic because the diffusion layer does not affect the internal structure of the OLEDs. On the other hand, in the luminance characteristics, the value of the OLEDs using the diffusion layer was higher than that of the OLED without the diffusion layer, in the order of Device C, Device B, Device A, and Non-Cavity. It can be seen from this luminance tendency that the OLED device with a high-refractive-index diffusion layer has a higher luminance value. As shown in Fig. 3(b), the OLEDs with a diffusion layer show higher efficiency than the devices without a diffusion layer, and Device C, which has the highest refractive index, has an efficiency value of 3.91 cd/A at 1000 cd/m^2^, which is 26.9% higher than that of the Non-Cavity device. The same tendency is seen in power efficiency and external quantum efficiency (EQE). In the power-efficiency curve according to the luminance, the Non-Cavity device, Device A, Device B, and Device C have power efficiency values of 0.94, 1.06, 1.15, and 1.26 lm/W at 1000 cd/m^2^, respectively. The EQE curves also showed efficiencies of 1.06, 1.21, 1.29, and 1.39% at 10 mA/cm^2^, respectively, and the efficiency of Device C using the ZnO refractive-index-matching layer was the highest. These efficiency-enhancement results demonstrate that the diffusion layer applied to the OLED has a reduced substrate mode due to a superior scattering effect and a rough outer surface. The EL characteristics of OLEDs with a diffusion layer are summarized in Table 1.

**Table 1:** Summary of characteristics of OLEDs and MC-OLEDs with diffusion layer.

	Current efficiency (cd/A)	Power efficiency (lm/W)	EQE (%)	Peak shift (0° → 60°)	Δ(u’,v’) (0° → 60°)
Non-Cavity^a^	3.08	0.94	1.06	22 nm	0.0142
Device A^a^	3.41	1.06	1.21	18 nm	0.0129
Device B^a^	3.65	1.15	1.29	8 nm	0.0087
Device C^a^	3.91	1.26	1.39	4 nm	0.008
Micro-Cavity^b^	4.53	1.3	1.4	18 nm	0.038
Device 1^b^	4.5	1.32	1.39	18 nm	0.034
Device 2^b^	4.17	1.2	1.46	14 nm	0.029
Device 3^b^	4.17	1.2	1.44	10 nm	0.027

^a^These devices measured current and power efficiency at 1000 cd/m^2^ and EQE at 10 mA/cm^2^. ^b^These devices measured current and power efficiency at 2000 cd/m^2^ and EQE at 50 mA/cm^2^.

**Figure 4:**
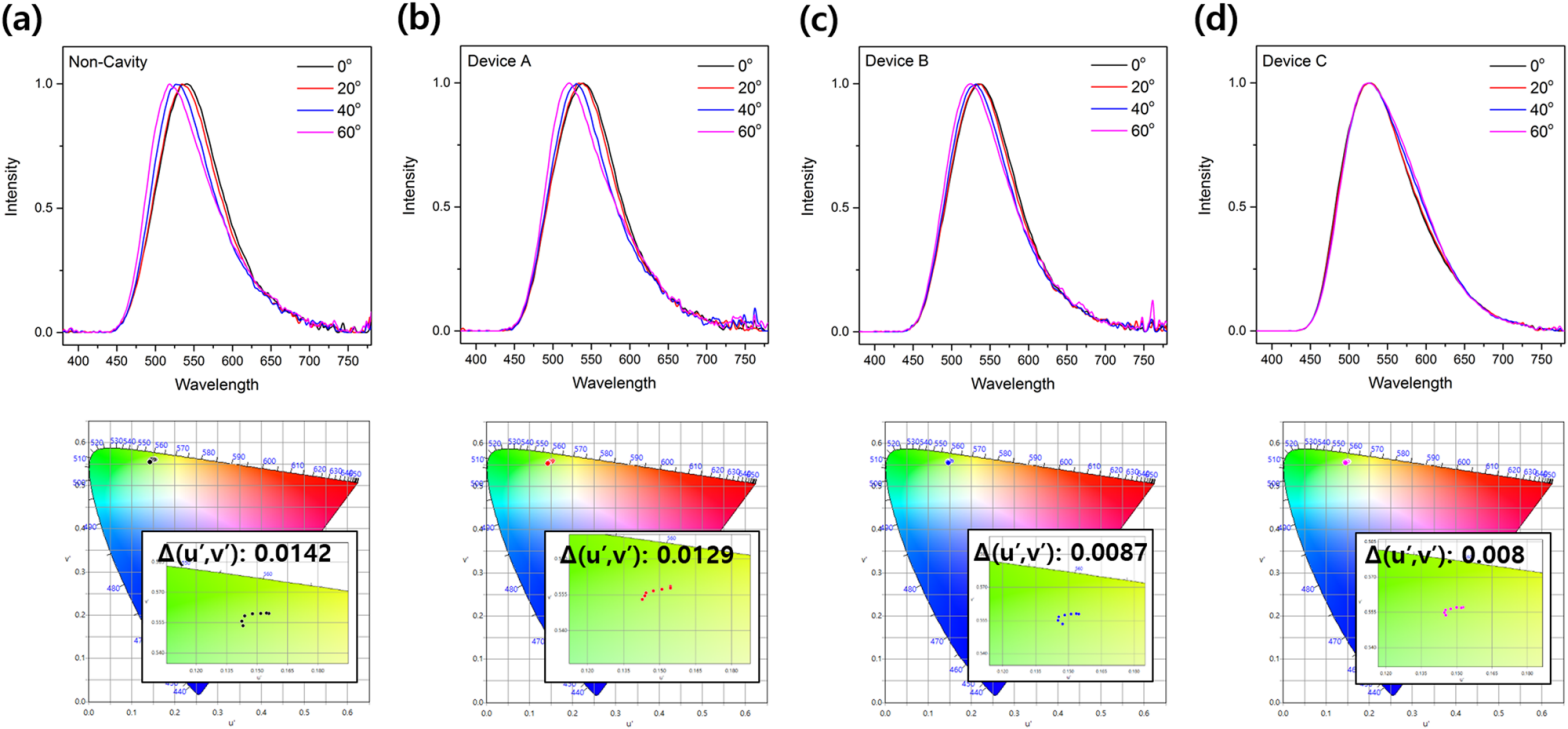
Viewing angle characteristics of the OLEDs with different diffusion layers (above: EL spectra, below: color coordinates according to viewing angle): (**a**) Non-Cavity device, (**b**) Device A (**c**) Device B, and (**d**) Device C.

Next, we analyzed the viewing angle characteristics of OLEDs using diffusion layers. Figure 4 shows the EL spectra and the color-coordinate shifts according to the viewing angle of each device. As the viewing angle changes from 0° to 60°, the peak of the EL spectra of all devices except for Device C was blue-shifted to the ultraviolet region. The Non-Cavity device without a diffusion layer shows a peak shift of 22 nm in total, but OLEDs with a diffusion layer have a shorter peak-shift distance of 18 nm, 8 nm, and 4 nm, respectively. This spectral-peak shifting tendency also shows that the higher the haze of OLED’s high-refractive-index diffusion layer, the shorter the shift distance of the EL-spectral peak. In the case of the color-coordinate shift according to the viewing angle shown in Fig. 4, the shift of the color coordinate according to the viewing angle decreases as the haze of the diffusion layer applied to the OLEDs increases (Non-Cavity: 0.0142, Device A: 0.0129, Device B: 0.0087, and Device C: 0.008). Devices A, B, and C with diffusion layers have a wider emission pattern, due to the scattering of light in the diffusion layer, than the Non-Cavity device, which has an angular emission pattern similar to Lambertian distribution. The angular emission pattern of these devices is shown in Fig. S2 (Supporting Information, Fig. S2).

In this chapter, we have confirmed that when a diffusion layer is applied to the conventional OLED, we can improve the light efficiency by reducing the substrate mode and further improve the color shift or spectral peak shift according to the viewing angle.

### Optical properties of diffusion layer in MC-OLEDs

In this chapter, we applied the previously fabricated diffusion layer to the MC-OLEDs with micro-cavity structure to see how the diffusion layer affects the device. As the anode of the MC-OLED, an ITO/Ag bilayer was used and the same organic materials as those in the conventional OLED made in previous chapters were used. The detailed MC-OLED structure is shown in Fig. S1 (Supporting Information, Fig. S1). The manufactured MC-OLEDs are classified as follows: **Micro-Cavity** (only MC-OLED), **Device 1** (MC-OLED with diffusion layer 1), **Device 2** (MC-OLED with diffusion layer 2), and **Device 3** (MC-OLED with diffusion layer 3). Because it is very important to optimize the cavity length, which is the thickness of the organic layers between Ag and Al in the MC-OLED, we calculated the cavity length with the abbreviated Fabry–Pérot interference–constructive-interference condition described by Eq. (2)^29^.

2 $${\rm{\Delta }}{\varnothing }_{{\rm{FP}}}={\varnothing }_{a}+{\varnothing }_{c}-\sum _{i={i}_{th}}\frac{4\pi {n}_{i}{d}_{i}cos\theta }{\lambda }=2\pi m,$$

where ${\rm{\Delta }}{\varnothing }_{{\rm{FP}}}$ is the phase term of the Fabry–Pérot interference and ${\varnothing }_{a}$ and ${\varnothing }_{c}$ are the phase changes that occur with the reflections at the anode/organic and cathode/organic interface, respectively. ${n}_{i}$ and ${d}_{i}$ are the refractive index and thickness of the ${i}_{th}$ layer, λ is wavelength of emitted light, and m is an integer.

**Figure 5:**
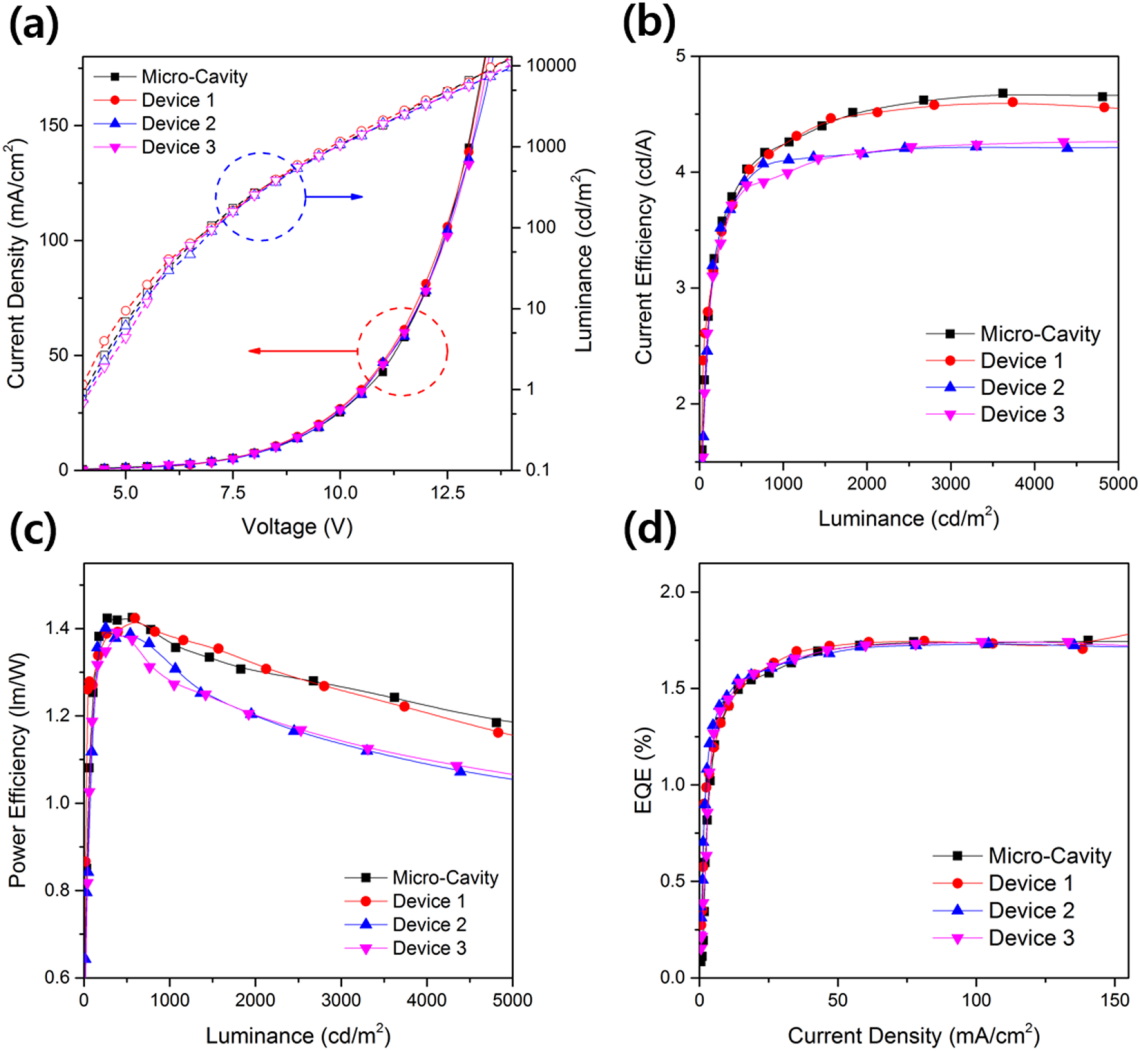
EL characteristics of MC-OLEDs with diffusion layers: (**a**) current-density–voltage-luminance, (**b**) current-efficiency–luminance, (**c**) power-efficiency–luminance, and (**d**) EQE–current-density relations.

The EL characteristics of the MC-OLEDs with the diffusion layer that is fabricated by applying the cavity length obtained by Eq. (2) are shown in Fig. 5. As with the conventional OLEDs of the above chapter, all MC-OLEDs have the same internal structure, so the current-density characteristics of the MC-OLEDs are similar for all devices. In the case of the luminance characteristics, the Micro-Cavity device without diffusion layer and Device 1 that has the diffusion layer with the lowest haze have similar luminance values, but Device 2 and 3, which have diffusion layers with relatively high haze values, have lower luminance than the Micro-Cavity device and Device 1. The luminance of the two devices is reduced because the normal-direction light enhanced by the resonance of the micro-cavity structure is scattered by the diffusion layer at various viewing angles. Due to the scattering effect of the diffusion layer, the current efficiency and power efficiency calculated by the measured light in the normal direction are similar to the luminance tendency. At a luminance of 2000 cd/m^2^, the devices have efficiency values of 4.53 cd/A, 4.5 cd/A, and 4.17 cd/A for Micro-Cavity, Device 1, and Devices 2 and 3, respectively. Similarly, each device has a power efficiency value of 1.3 lm/W, 1.32 lm/W, and 1.2 lm/W for Micro-Cavity, Device 1, and Devices 2 and 3, respectively, at 2000 cd/m^2^. However, the EQE recalculated through the angular emission pattern measured every 10° shows a different tendency from the current and power efficiency. As can be seen in Fig. 5(d), all MC-OLEDs have similar EQE values. This EQE characteristic shows that the normal-direction lights were improved due to the dispersal in the micro-cavity structure by the diffusion layer. The light intensity decreases when measured from the normal direction, but the total light intensity combining all the lights scattered at different angles is not changed. In other words, the diffusion layer applied to the outside of the MC-OLEDs can effectively scatter the light at various angles without loss of total light quantity. The EL characteristics of MC-OLEDs with a diffusion layer are summarized in Table 1.

**Figure 6:**
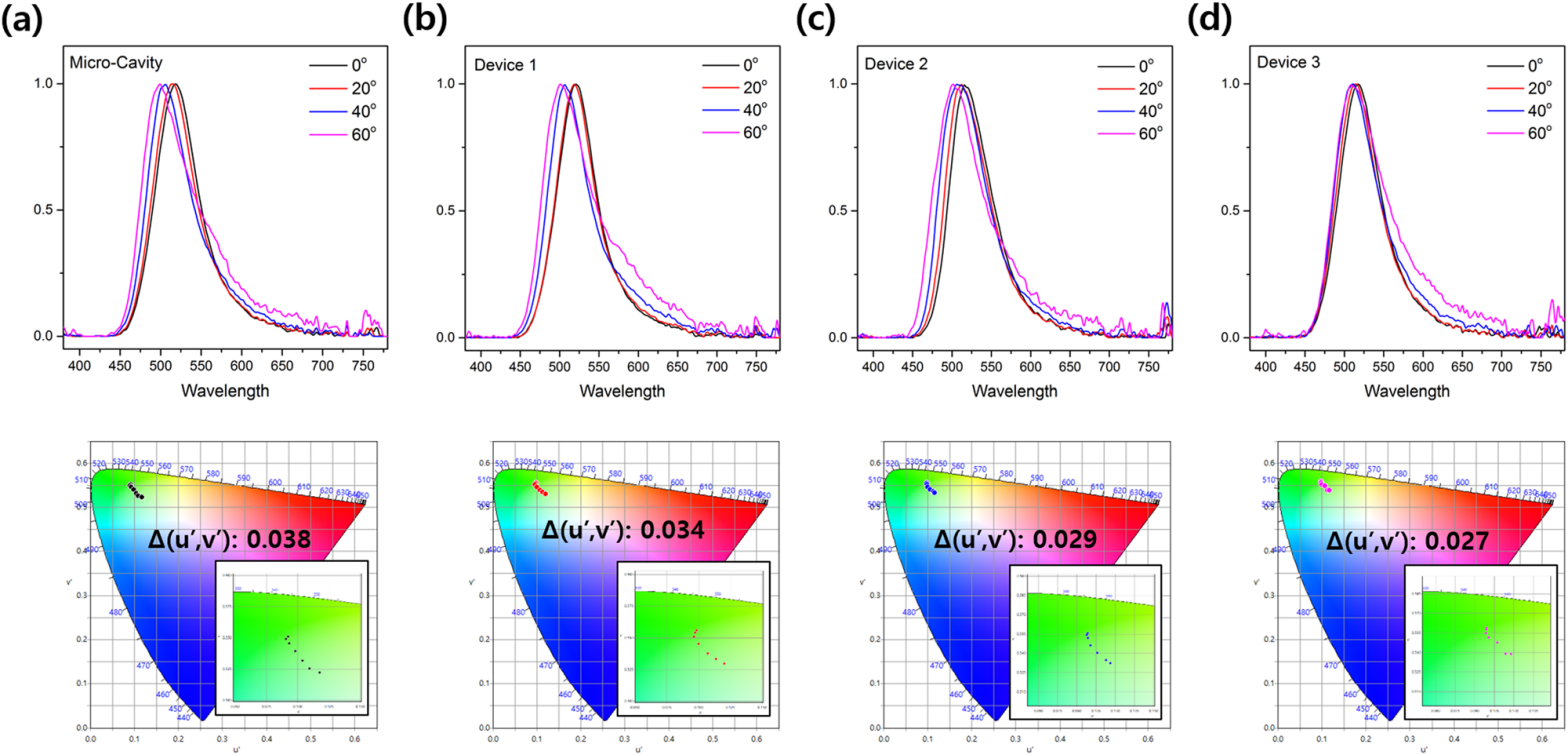
Viewing angle characteristics of the OLEDs with different diffusion layers (above: EL spectra, below: color coordinate according to viewing angle): (**a**) Micro-Cavity device, (**b**) Device 1, (**c**) Device 2, and (**d**) Device 3.

Next, we examined how to improve the viewing-angle-dependent characteristics of MC-OLEDs by applying a diffusion layer. As shown in Fig. 6, the blue-shift of the EL spectra of the device decreases with the application of the diffusion layer as the viewing angle changes. In addition, because the devices have a micro-cavity resonance structure, it can be confirmed that the full width at half maximum (FWHM) of the spectra is narrower than that of the EL spectra of the above chapter OLEDs. The EL spectra of the Micro-Cavity device without the diffusion layer and Device 1 with a low-haze diffusion layer both shift 18 nm, and the spectra of Devices 2 and 3 shift 14 and 10 nm, respectively. In the case of the color-coordinate shift according to the viewing angle shown in Fig. 6. The shift of the color coordinate according to the viewing angle decreases as the haze of the diffusion layer applied to the MC-OLEDs increases (Micro-Cavity: 0.038, Device 1: 0.034, Device 2: 0.029, and Device 3: 0.027). Moreover, all devices have a micro-cavity structure, so they have a narrower angular emission pattern than the Lambertian distribution. However, Devices 1, 2, and 3 with diffusion layers have a wider emission pattern, due to the scattering of light in the diffusion layer, than the Micro-Cavity device without a diffusion layer, and as the haze of the diffusion layer of the MC-OLEDs increases, its angular emission pattern becomes close to the Lambertian distribution. The angular emission pattern of MC-OLEDs is shown in Fig. S3 (Supporting Information, Fig. S3).

### Reflectance of diffusion layer with polarizer film

All OLED display products use polarizer film to prevent significant contrast-ratio loss due to reflection of external light in bright indoor or outdoor conditions. To verify whether the contrast ratio was reduced by diffusion layers in bright conditions, we measured the reflectance of four samples with polarizer film on the diffusion layer as follows:

**Ag**: Glass/Ag (100 nm)

**Sample A**: Polarizer film/Glass/Ag (100 nm)

**Sample B**: Polarizer film/diffusion layer 1/glass/Ag (100 nm)

**Sample C**: Polarizer film/diffusion layer 2/glass/Ag (100 nm)

**Sample D**: Polarizer film/diffusion layer 3/glass/Ag (100 nm)

**Figure 7:**
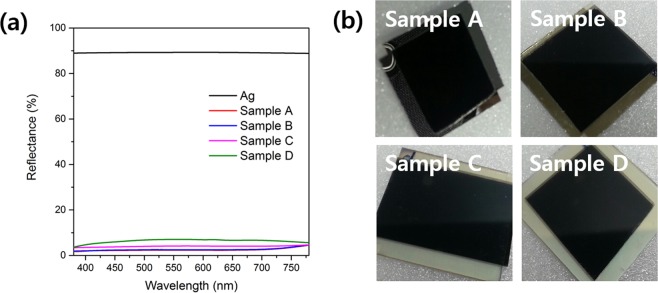
(**a**) The reflectance of various samples and (**b**) black color of various samples.

Figure 7 shows the reflectance of each sample and the black color of the samples through polarizer film. As shown in Fig. 7(a), Sample A, Sample B, Sample C, and Sample D have average reflectance values of 2.47%, 2.63%, 4.02%, and 6.35%, respectively. The low reflectance of Sample A is due to the external light being linearly polarized through the polarizer film and reflected by the Ag to be rotated by 90° so that it is blocked by the polarizer film. Samples B, C, and D, which have a diffusion layer, slightly increased in reflectance compared to Sample A with only polarizer film. In particular, the degree of increase of the reflectance increased with the scattering degree of the diffusion layer. However, in the black color of each sample in Fig. 7(b), it can be seen that the samples with slightly increased reflectance due to the diffusion layer do not have any problem in producing a black color. This result means that the contrast-ratio degradation is not serious when using polarizer film with a diffusion layer in a bright indoor/outdoor environment because the reflectance increase due to the diffusion layer is not critical.

In conclusion, we fabricated a bilayer-type diffusion layer with different refractive indices to improve the light efficiency and viewing-angle characteristics of OLED devices. The fabricated diffusion layer had an increasing haze value as the refractive index difference between the two layers increased. When the diffusion layer is applied to the device, the light efficiency and viewing-angle characteristics are improved in the conventional OLEDs and in the MC-OLEDs, and the viewing-angle characteristics are effectively improved without any light loss. In addition, the black-color implementation of the device when the diffusion layer was used with the polarizer film was found to be insignificantly affected by the diffusion layer due to the black color of the device. These results demonstrate that diffusion layers are effective in fabricating OLED displays with high efficiency and viewing-angle independence.

## Methods

### Diffusion layer

The diffusion layer is composed of a refractive-index-matching layer of a PMMA nanostructure and metal oxide. The process of the PMMA nanostructure is as follows. First, ITO-coated glass substrates (Corning, Inc.) were immersed in acetone, methanol, and deionized water and then cleaned in an ultrasonic bath for 15 minutes. When the cleaning step was finished, the substrates were blown with nitrogen and the residual solution on the surface of the substrate was shaken off. The substrates were then dried in an oven at 110 °C. Then, PMMA (495 PMMA A8, Microchem Co.) was sprayed 600 μl from the opposite side of ITO glass substrate (with no ITO side) with a micropipette and coated on the glass by spin coating. The spin-coating step is performed sequentially at 500 rpm for 1 s and 5 s, and at 1000 rpm for 1 s and 45 s. The ITO glass substrate coated with PMMA was heated at 170 °C for 40 min on a hotplate to cure the PMMA, and then dry etching with plasma energy using an RIE to produce a diffusion layer with nanoscale structure. The plasma gas used for dry etching was sequentially used with O_2_. The RIE was carried out for 2 min with RF power of 200 W. Al_2_O_3_ and ZnO in the refractive-index-matching layer on the PMMA nanostructures were deposited using RF sputtering. During the sputtering, Ar gas was introduced to form a plasma, and RF power of 150 W was used. This process was carried out under a vacuum of 4 × 10^−5^ Torr and each refractive-index-matching layer was deposited to a thickness of 70 nm.

### Fabrication device

OLEDs and MC-OLEDs were sequentially deposited on the ITO of the ITO–glass substrate on which the diffusion layer was formed. All organic and metal layers were deposited by thermal evaporation under a vacuum of 3 × 10^−6^ Torr. In the OLEDs with an ITO anode, 5 nm of HATCN was used as a hole-injection layer, 50 nm of NPB was used as a hole-transport layer, 80 nm of Alq_3_ was used as an emitting layer and electron-transport layer, 0.5 nm of LiF was used as an electron-injection layer, and 100 nm of Al was used as a cathode. Then, in the MC-OLEDs with an ITO/Ag anode, 5 nm of HATCN was used as a hole-injection layer, 50 nm of NPB was used as a hole-transport layer, 55 nm of Alq_3_ was used as an emitting layer and electron-transport layer, 0.5 nm of LiF was used as an electron-injection layer, and 100 nm of Al was used as a cathode. All organics were deposited at a rate of 0.5–1 Å/s and the metal was deposited at a rate of 1–3 Å/s, measured by a quartz crystal sensor in the chamber.

### Measurement

The surface morphologies of the PMMA nanoscale structure and diffusion layer were measured using FE-SEM (S-4800, Hitachi High-Technologies, Inc.). The optical transmittance and reflectance were measured with a UV-vis spectrometer (Cary-5000, Agilent Technologies, Inc.) and the EL characteristics of the OLED devices were measured using a spectroradiometer (Spectra Scan PR-670, Photo Research, Inc.) in a dark box with a source meter (Model 237, Keithley Instruments, Inc.) without any encapsulation.

## Supplementary information

Related Manuscript File
