# Supplementary material for: Optical characteristics of refractive-index-matching diffusion layer in organic light-emitting diodes
Source: Sci Rep. 2019 Jun 18;9:8690. doi: 10.1038/s41598-019-45000-w (PMC6581959; doi:10.1038/s41598-019-45000-w)
Supplement: Supplementary file 1 — Related Manuscript File [file 41598_2019_45000_MOESM1_ESM.docx]

**Supporting Information**

**Optical characteristics of refractive-index-matching diffusion layer in organic light-emitting diodes**

**Cheol Hwee Park^1,§^, Jae Geun Kim^1,§^, Sun-Gyu Jung^1^, Dong Jun Lee^1^, Young Wook Park^2,*^, and Byeong-Kwon Ju^1,*^**

^§^ These authors contributed equally to this work.

^1^Display and Nanosystem Laboratory, School of Electrical Engineering, Korea University Seoul 136-713, Republic of Korea

^2^School of Mechanical and ICT Convergence Engineering, SUN MOON University, Chungcheongnam-do 31460, Republic of Korea

^*^Correspondence and requests for materials should be addressed to Y.W.P. (email: zerook@sunmoon.ac.kr) or B.-K.J. (email: bkju@korea.ac.kr)

Phone No.: +82-2-3290-3665

Fax. No.: +82-2-3290-3791

**Table. S1 Total transmittance and haze of diffusion layer with various refractive index**

|  | **Diffusion layer 1**  **R.I=1.5 (PMMA)** | | **Diffusion layer 2**  **R.I=1.75 (PMMA+Al_2_O_3_)** | | **Diffusion layer 3**  **R.I=2.0 (PMMA+ZnO)** | |
| --- | --- | --- | --- | --- | --- | --- |
|  | **Total T** | **Haze** | **Total T** | **Haze** | **Total T** | **Haze** |
| **at 540nm** | **91.9** | **8.6** | **87.2** | **22.2** | **86.1** | **30.5** |


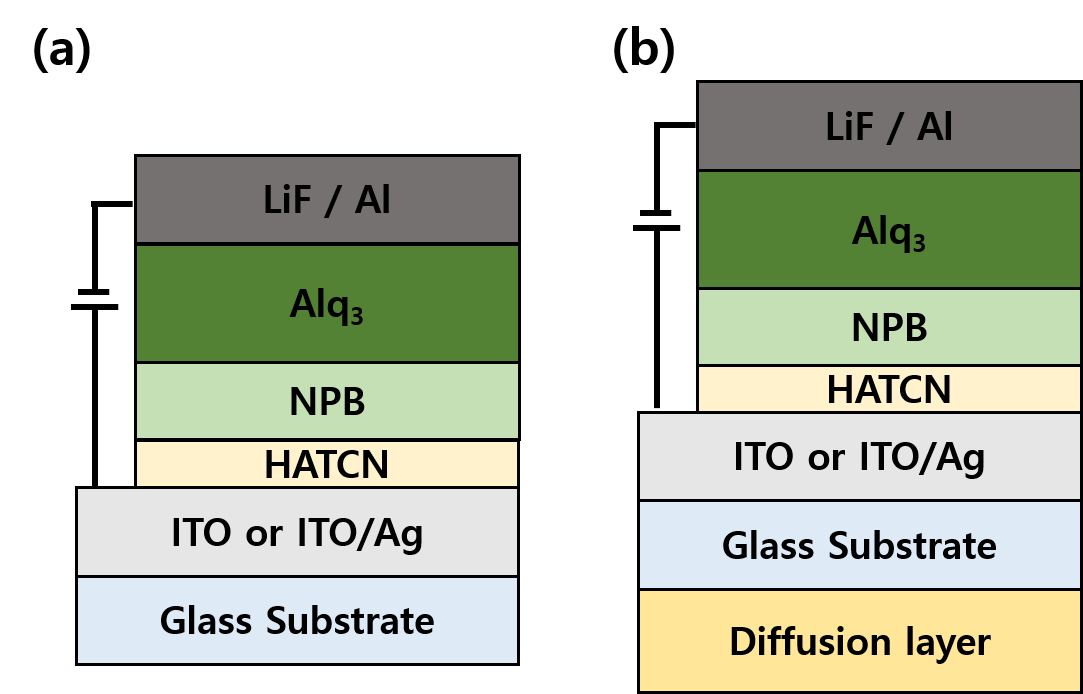


**Supplementary Figure S1**. The detailed structure of OLEDs and MC-OLEDs with diffusion layer


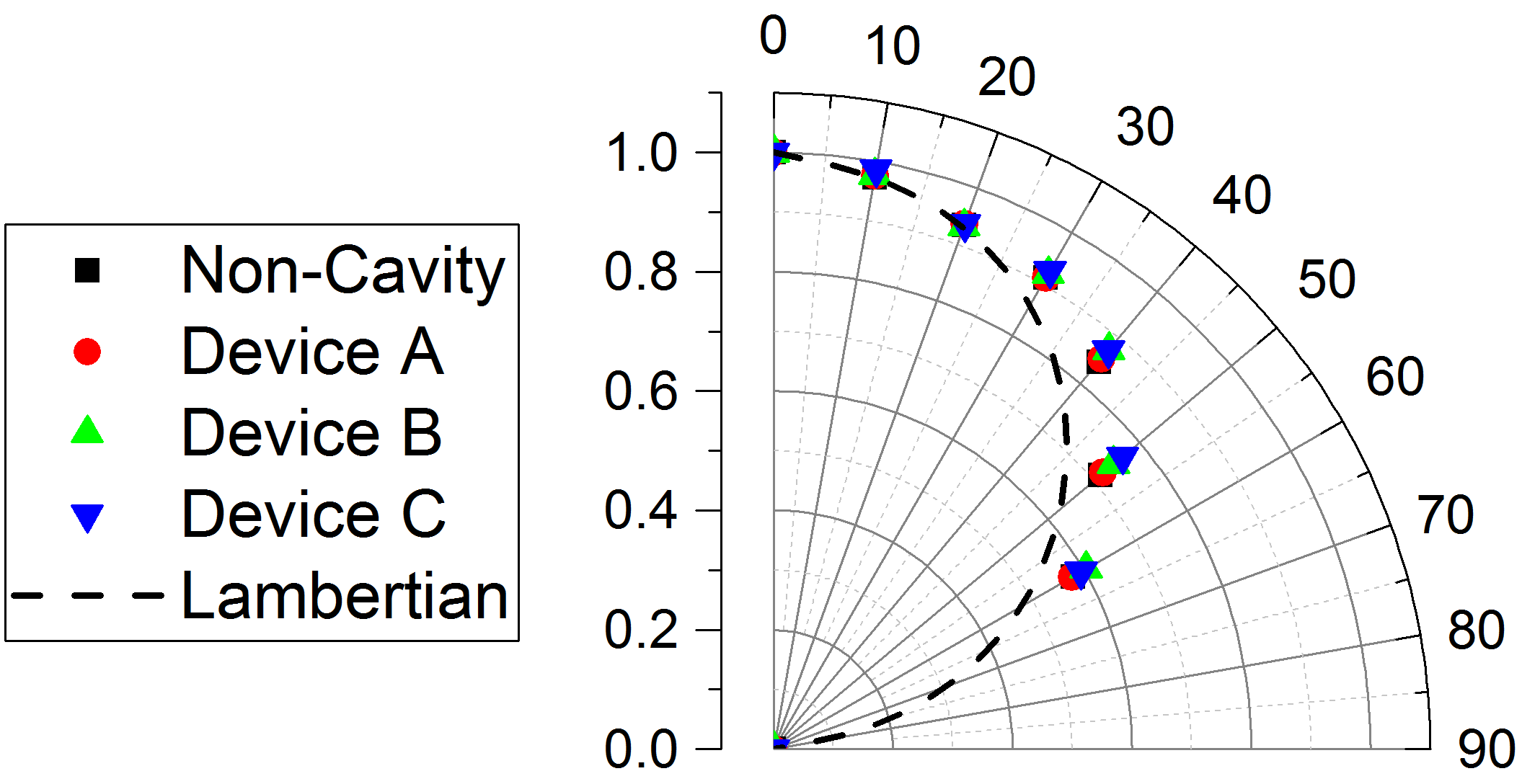


**Supplementary Figure S2**. The angular emission pattern of OLEDs with diffusion layer


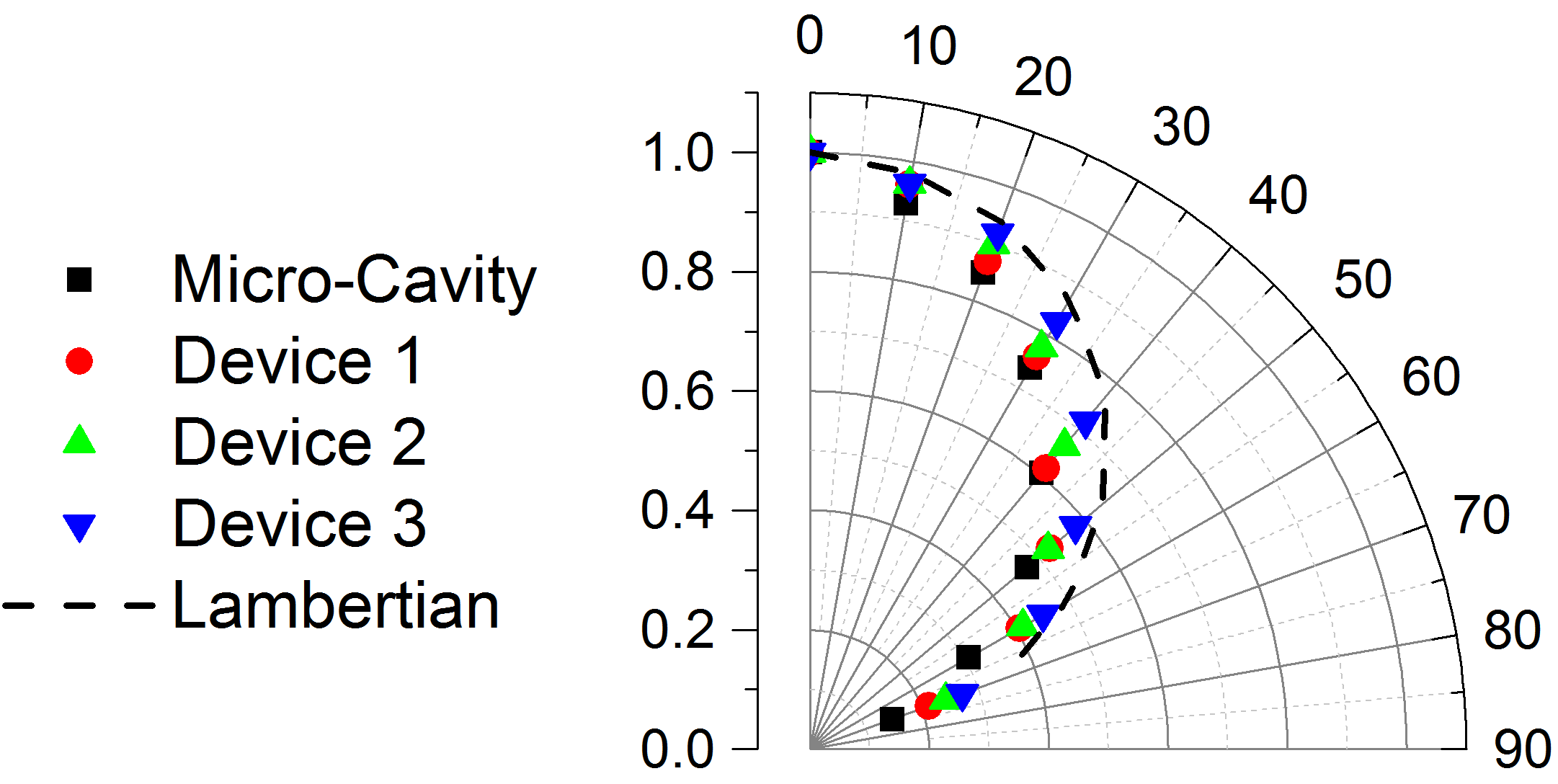


**Supplementary Figure S3**. The angular emission pattern of MC-OLEDs with diffusion layer
